# Soy Consumption and the Risk of Prostate Cancer: An Updated Systematic Review and Meta-Analysis

**DOI:** 10.3390/nu10010040

**Published:** 2018-01-04

**Authors:** Catherine C. Applegate, Joe L. Rowles, Katherine M. Ranard, Sookyoung Jeon, John W. Erdman

**Affiliations:** 1 Division of Nutritional Sciences, University of Illinois at Urbana-Champaign, Urbana, IL 61801, USA; cca2@illinois.edu (C.C.A.); jrowles2@illinois.edu (J.L.R.); ranard2@illinois.edu (K.M.R.); sjeon17@illinois.edu (S.J.); 2 Department of Food Science and Human Nutrition, University of Illinois at Urbana-Champaign, Urbana, IL 61801, USA

**Keywords:** prostate cancer, soy, isoflavones, epidemiology, cohort, case-control

## Abstract

Prostate cancer (PCa) is the second most commonly diagnosed cancer in men, accounting for 15% of all cancers in men worldwide. Asian populations consume soy foods as part of a regular diet, which may contribute to the lower PCa incidence observed in these countries. This meta-analysis provides a comprehensive updated analysis that builds on previously published meta-analyses, demonstrating that soy foods and their isoflavones (genistein and daidzein) are associated with a lower risk of prostate carcinogenesis. Thirty articles were included for analysis of the potential impacts of soy food intake, isoflavone intake, and circulating isoflavone levels, on both primary and advanced PCa. Total soy food (*p* < 0.001), genistein (*p* = 0.008), daidzein (*p* = 0.018), and unfermented soy food (*p* < 0.001) intakes were significantly associated with a reduced risk of PCa. Fermented soy food intake, total isoflavone intake, and circulating isoflavones were not associated with PCa risk. Neither soy food intake nor circulating isoflavones were associated with advanced PCa risk, although very few studies currently exist to examine potential associations. Combined, this evidence from observational studies shows a statistically significant association between soy consumption and decreased PCa risk. Further studies are required to support soy consumption as a prophylactic dietary approach to reduce PCa carcinogenesis.

## 1. Introduction

Prostate cancer (PCa) is the second most commonly diagnosed cancer in men worldwide. According to the International Agency for Research on Cancer’s GLOBOCAN database, 1.1 million men were diagnosed with PCa in 2012, accounting for 15% of all cancers in men [1]. Incidence rates are lowest in Asian countries, where soy foods are regularly consumed as part of a normal diet. Several studies have reviewed the inverse association seen between soy food intake and PCa incidence in Asian populations, proposing that soy isoflavones act as weak hormones to exert a protective physiological effect against the development of PCa [2,3,4,5]. Indeed, the soy isoflavones, genistein and daidzein, have been shown to accumulate in prostatic tissue [6], where they may be cytotoxic to cancer cells [7]. These effects may occur as a result of both non-hormonal and hormonal action. For example, genistein upregulates tumor suppressor genes in PCa cells [8] and suppresses prostate carcinogenesis in an estrogen receptor (ER) wild-type mouse model, when compared to ER knock-out mouse models [9].

This hypothesis has further been supported by four previous meta-analyses of epidemiological studies, all of which showed a protective association between soy consumption and PCa [10,11,12,13]. However, these meta-analyses did not integrate and evaluate all available existing studies pertaining to both dietary soy food intake and circulating levels of isoflavones. Our current analysis broadens the included categories of soy food and isoflavone measurements, allowing for a more complete review of the literature. We include a larger number of studies and an in-depth analysis on the relationship between total soy food intake, fermented and unfermented soy food intakes, individual and combined dietary isoflavones, and circulating individual and combined isoflavones and the risk of PCa. Furthermore, no previous meta-analysis has explored potential links between soy foods and advanced PCa. Because soy isoflavones have also been linked to inhibiting PCa cell motility and invasion [14] and reducing inflammatory markers in men with PCa [15], we also evaluated the impact of soy on the risk of advanced PCa. 

## 2. Materials and Methods

### 2.1. Study Selection Criteria

This meta-analysis was conducted following PRISMA and Meta-analysis of Observational Studies in Epidemiology (MOOSE) guidelines [16,17]. Studies fitting the following criteria were included in this meta-analysis: (a) examined the relationship between soy and PCa risk by using randomized control trials and/or cohort, cross-sectional, retrospective, prospective and case-control studies; (b) methodology was reported in replicable detail; (c) examined the association between soy and PCa risk; (d) reported relative risk ratios with 95% confidence intervals for the reported exposure categories; (e) were written in English; and (f) were peer-reviewed publications. 

### 2.2. Literature Search

We conducted a thorough literature search of PubMed, Web of Science, and the Cochrane Library, using a combination of the following key words and their variants: prostate cancer, prostate neoplasm, soy, soymilk, soy milk, isoflavone, bean curd, tofu, soy protein, daidzein and genistein (up to 25 May 2017). The keyword search yielded a comprehensive list of titles and abstracts of articles that were screened for relevance against the listed study selection criteria. These screened articles were evaluated in full text for study inclusion. Finally, we conducted a reference list search (i.e., backward search) and works cited search (i.e., forward search) from the articles identified as meeting the inclusion criteria. The studies identified through this process were further assessed using the same inclusion criteria, with this process being repeated until all relevant articles were identified. Three authors (CA, JR3 and KR) individually considered all articles obtained in full text for inclusion or exclusion, and any discrepancies were discussed and resolved. 

### 2.3. Data Extraction and Quality Assessment

The following information was extricated from each article: name of first author; year of publication; location of study; study period; number of cases, controls and total number of participants in the study; age of participants; total years of follow-up; exposure values of soy (serum/plasma and/or intake); relative risk ratios for PCa; adjustments made for any covariates; and study type. The term RR (relative risk) will be used in this study as a general term to denote the following: relative odds (cumulative incidence data), rate ratio (incidence-rate data) and odds ratios (OR; case-control data). Study quality was examined using the Newcastle–Ottawa Scale, which is a validated scale used to assess the quality for case-control and non-randomized cohorts in a meta-analysis [18]. This scale evaluates each study based on the following three categories: selection of cases and controls, comparability of studies, and exposure of the main variable (soy, genistein, daidzein, or total isoflavones). We regarded scores of 1–3, 4–6 and 7–9 as low, medium and high quality, respectively. The resulting quality score was included as a measurement of the strength of the evidence presented in each study and was not used to determine the inclusion or exclusion of studies. The significance of the study quality was analyzed in the subgroup analysis. 

### 2.4. Statistical Analysis

STATA/IC version 14.2 (StataCorp LP, College Station, TX, USA) was used to analyze the data. Because OR is nearly equivalent to RR when considering low incidence of diseases [19], RR and 95% confidence intervals (CI) were used as a measure of the effect size for all included studies. Heterogeneity amongst studies was determined using the *I*^2^ statistic [20]. Fixed and random (DerSimonian–Laird) effects models were used, depending on the *I*^2^ result, as markers of study heterogeneity [20]. An *I*^2^ value of less than 50% (*I*^2^ < 50%) signified low-to-moderate heterogeneity between studies, so a fixed effect model was used to determine RR estimates. An *I*^2^ value of greater than or equal to 50% (*I*^2^ ≥ 50%) signified moderate-to-substantial heterogeneity between studies, so a random effects model was used to determine RR estimates. When results from fixed and random effects models were conflicting and studies showed moderate heterogeneity (*I*^2^ = 30–60%), we presented the latter as it represents a more conservative approach [21,22]. 

Because studies reported different exposure categories as tertiles, quartiles or quintiles, we used the study specific RR for the highest quantile of dietary soy intake and/or circulating (serum or plasma) isoflavone concentrations. Potential publication bias was assessed by using funnel plots [23,24], Egger’s linear regression test [25], and Begg’s rank correlation test of asymmetry [26]. We also performed sensitivity analyses to evaluate whether the pooled results could have been affected by excluding a single study at a time. Subgroup analyses were performed on study type, study location, study quality, and individual covariate adjustment. A *p*-value of less than 0.05 was considered statistically significant for all analyses.

## 3. Results

### 3.1. Literature Search

**Figure 1:**
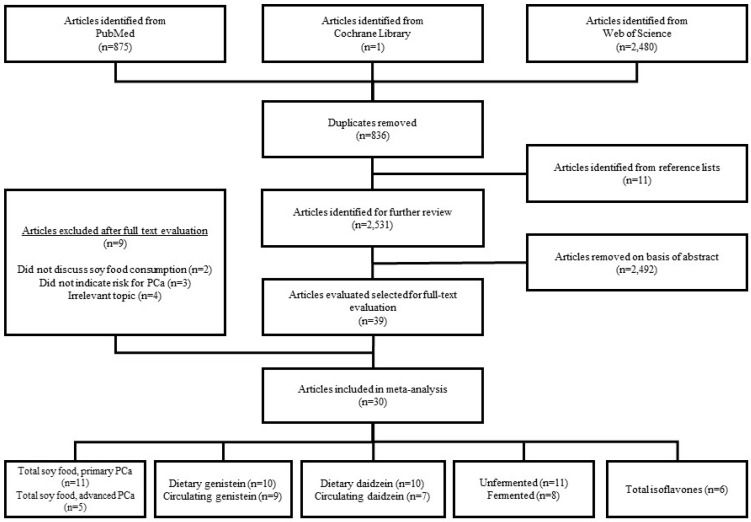
Literature search and study selection flow chart.

In total, 3356 articles were identified from the library search engines. After removing duplicates and adding articles identified from reference lists, 2531 articles remained. Of the 2531 articles that were screened by abstract, 39 articles were found to contain potentially relevant information and were evaluated by full text review. Upon reviewing the full text articles for the aforementioned inclusion criteria, 30 articles were included in the final analysis [27,28,29,30,31,32,33,34,35,36,37,38,39,40,41,42,43,44,45,46,47,48,49,50,51,52,53,54,55,56]. Of these 30 articles, 24 [27,28,29,30,31,32,33,34,36,37,38,39,40,41,42,43,45,46,47,48,49,52,53,55] included information regarding dietary soy intake and nine [29,35,40,42,44,50,51,54,56] included information regarding circulating isoflavone levels (Figure 1).

### 3.2. Study Characteristics

Of the 30 articles included for analysis, fifteen [28,29,30,32,34,37,38,39,41,42,47,48,49,52,56] articles were case-control studies, eight [27,31,33,36,43,45,46,53] articles were cohort studies, and seven [35,40,44,50,51,54,55] articles were nested case-control studies (NCC). The total number of study participants included was 266,699, and the total number of PCa cases reported was 21,612. Twelve [27,32,35,36,37,39,41,42,44,47,49,56] articles reported data from Asia, ten [31,33,34,38,43,45,46,48,52,53] articles reported data from North America, and eight [28,29,30,40,50,51,54,55] articles reported data from Europe. The study characteristics are summarized in Table 1. Articles analyzing soy intake from the diet used lifestyle questionnaires or validated food frequency questionnaires (FFQs) to collect usual dietary intake. All articles reported results using risk estimates as RR or OR. 

Quality scores were assigned to each article using the criteria outlined by the Newcastle–Ottawa scales for case-control and cohort studies. The average score for case-control studies was 6.93 (standard deviation = 0.7); 6 was the lowest score and 8 was the highest score given. Cohort and NCC studies received an average score of 7.73 (standard deviation = 0.7); 6 was the lowest score and 9 was the highest score given. Individual quality assessment scores are provided in Appendix A (Table A1).

### 3.3. Soy Intake and PCa Risk

**Figure A1:**
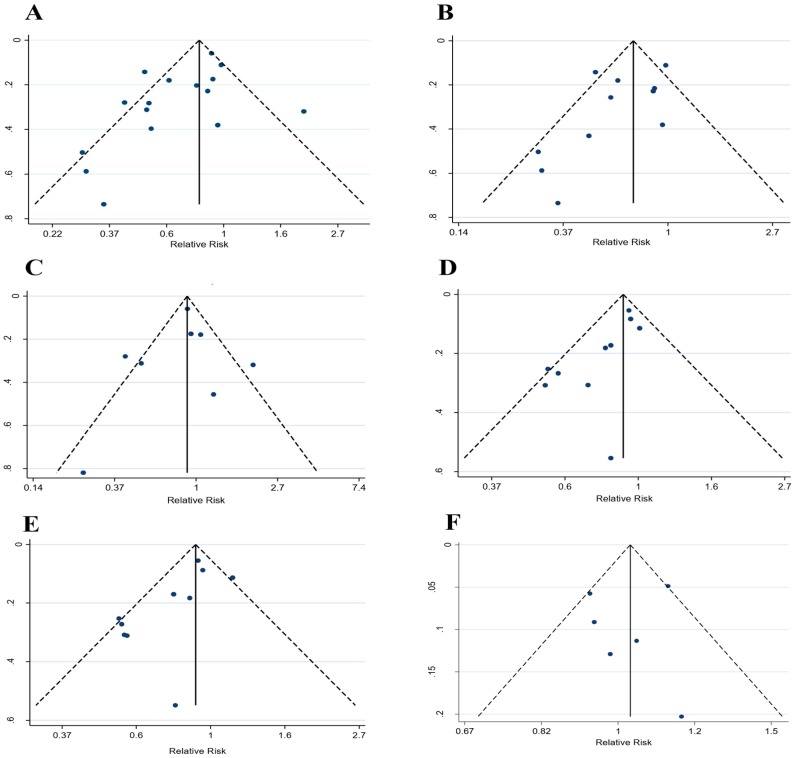
Funnel plots for (**A**) total soy intake and risk of prostate cancer; (**B**) unfermented soy intake and risk of prostate cancer; (**C**) fermented soy intake and risk of prostate cancer; (**D**) genistein intake and risk of prostate cancer; (**E**) daidzein intake and risk of prostate cancer; and (**F**) total isoflavone intake and risk of prostate cancer.

Twenty-four articles evaluated dietary soy and soy isoflavone intake and PCa risk. These articles were further divided into groups that analyzed risk pertaining to total soy (*n* = 16) [27,29,31,32,33,34,36,37,39,41,43,45,46,47,49,52], unfermented soy food (*n* = 11) [27,31,33,34,37,39,43,46,47,49,52], fermented soy food (*n* = 8) [27,32,36,37,41,45,46,47], genistein (*n* = 10) [30,36,37,38,40,41,42,45,48,55], daidzein (*n* = 10) [30,36,37,38,40,41,42,45,48,55], total isoflavones (*n* = 6) [28,29,30,45,53,55], tofu (*n* = 5) [27,37,43,46,47], miso (*n* = 3) [27,36,46], soy milk (*n* = 2) [31,49], and natto (*n* = 1) [47] intakes. Funnel plots used to explore publication bias are shown in Appendix A (Figure A1).

**Figure 2:**
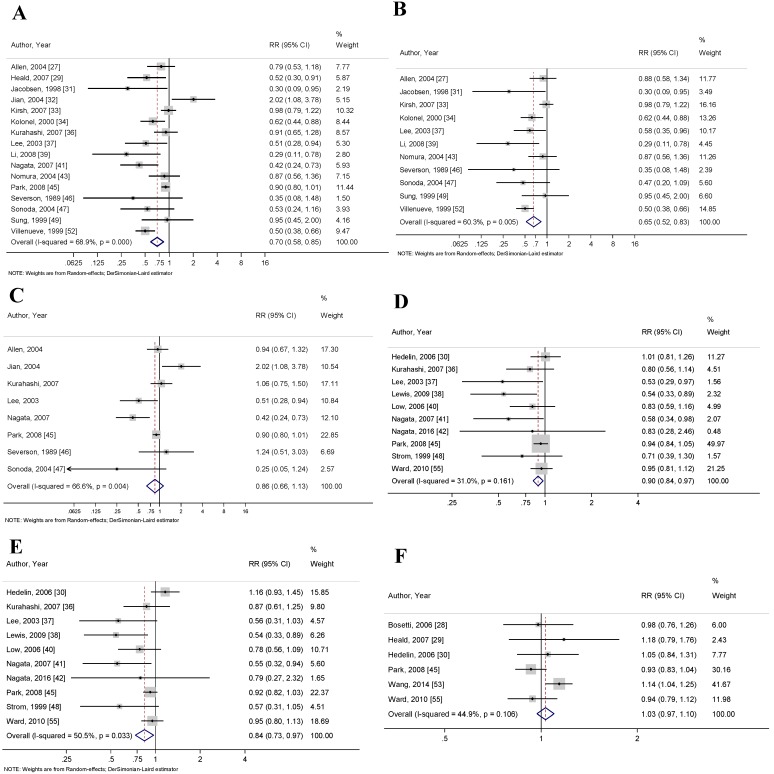
Forest plots for (**A**) total soy intake and risk of prostate cancer; (**B**) unfermented soy intake and risk of prostate cancer; (**C**) fermented soy intake and risk of prostate cancer; (**D**) genistein intake and risk of prostate cancer; (**E**) daidzein intake and risk of prostate cancer; and (**F**) total isoflavone intake and risk of prostate cancer. These associations were indicated as a relative risk (RR) estimate with the corresponding 95% confidence interval (CI).

Articles that reported soy intake as either a combination of multiple soy food items or as a single soy food item were classified as total soy intake. Sixteen articles reported the association between total soy intake and PCa risk. The pooled RR for this association was 0.71 (95% CI: 0.58–0.85, *p* < 0.001) (Figure 2A). Neither Begg’s correlation test (*p* = 0.300) nor Egger’s linear regression test (*p* = 0.052) for bias were significant. Heterogeneity amongst studies was analyzed using the *I*^2^ index to show high variation between studies (68.9%).

When selecting articles for evaluating the association between unfermented or fermented soy food intake and PCa risk, studies had to have explicitly stated which soy food items were being reported. Examples of commonly reported unfermented soy foods included soy milk, tofu and soybeans; fermented soy foods included miso and natto. The pooled RR for unfermented soy foods and risk of PCa was 0.65 (95% CI: 0.56–0.83, *p* < 0.001), and the pooled RR for fermented soy foods and risk of PCa was 0.86 (95% CI: 0.66–1.13, *p* = 0.218) (Figure 2B,C, respectively). Neither Begg’s correlation test (*p* = 0.161 and *p* = 0.902, respectively) nor Egger’s linear regression test (*p* = 0.117 and *p* = 0.670, respectively) for bias were significant. The *I*^2^ index showed high heterogeneity amongst studies included in the unfermented (60.3%) and fermented (66.6%) groups.

Ten articles reported soy intake as a measurement based on the calculation of genistein and daidzein present in soy foods. The pooled RR for genistein and risk of PCa was 0.90 (95% CI: 0.84–0.97, *p* = 0.008), and the pooled RR for daidzein and risk of PCa was 0.84 (95% CI: 0.73–0.97, *p* = 0.018) (Figure 2D,E, respectively). Begg’s correlation test was significant for genistein but was not significant for daidzein (*p* = 0.049 and *p* = 0.210, respectively), and Egger’s linear regression test was significant for both measurements (*p* = 0.009 and *p* = 0.039, respectively). The *I*^2^ index showed moderate heterogeneity between studies included in the genistein (31.0%) and daidzein (50.5%) groups.

Six articles reported isoflavone intake without disclosing the sources of the isoflavones. These studies were analyzed separately, so as not to interfere with measurements based solely on soy intake because isoflavones are found in other food items, such as seed sprouts and pulses. The pooled RR for isoflavone intake and PCa risk was 1.03 (95% CI: 0.97–1.09, *p* = 0.313) (Figure 2F). Neither Begg’s correlation test (*p* = 0.707) nor Egger’s linear regression test (*p* = 0.802) for bias were significant. The *I*^2^ index showed moderate heterogeneity between studies (44.9%). Notably, the inclusion of these studies in the total soy analysis did not significantly change the RR of dietary soy intake and PCa risk.

Finally, articles were further stratified into specific soy food groups, which included tofu, miso and soy milk. Few studies were available to accurately represent meta-analysis data on these points, so RR are reported here, but not included in further subgroup analyses. The pooled RR for tofu and PCa risk was 0.73 (95% CI: 0.57–0.94, *p* = 0.013), the pooled RR for miso and PCa risk was 1.01 (95% CI: 0.80–1.28, *p* = 0.919), and the pooled RR for soy milk and PCa risk was 0.58 (95% CI: 0.19–1.78, *p* = 0.343). None of the groups were significant for bias using Begg’s correlation test (*p* = 0.221, *p* = 0.296, and *p* = 1.000, respectively) or Egger’s linear regression test (*p* = 0.093, *p* = 0.497, and *p* = NA, respectively). The *I*^2^ index showed low heterogeneity amongst the studies included in the tofu (4.5%) and miso (0.0%) groups and high heterogeneity amongst studies included in the soy milk (63.1%) group.

### 3.4. Circulating Isoflavones and PCa Risk

**Figure 3:**
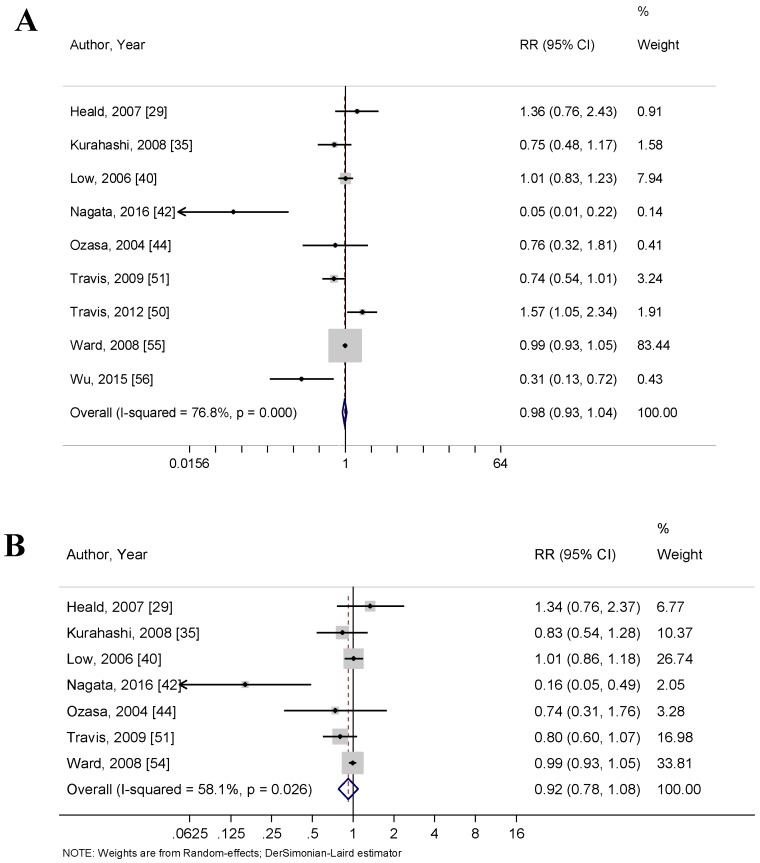
Forest plots for (**A**) circulating genistein and risk of prostate cancer; and (**B**) circulating daidzein and risk of prostate cancer. These associations were indicated as a relative risk (RR) estimate with the corresponding 95% confidence interval (CI).

**Figure A2:**
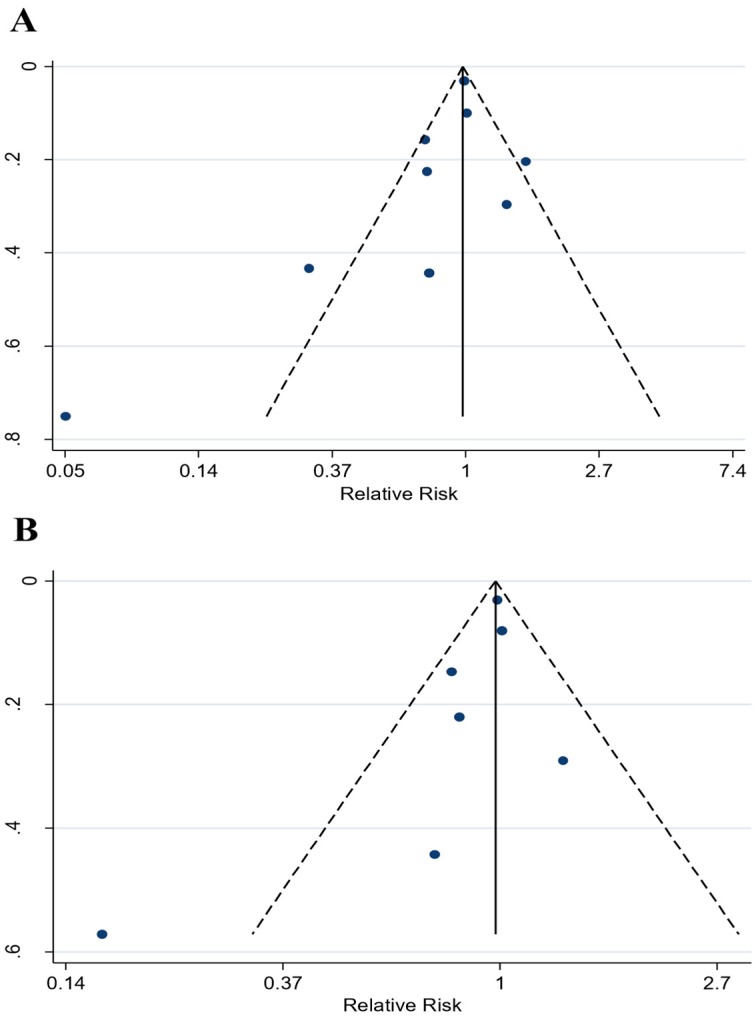
Funnel plots for (**A**) circulating genistein and risk of prostate cancer and (**B**) circulating daidzein and risk of prostate cancer.

Nine articles measured circulating isoflavone concentrations and their associations with PCa risk. Specifically, these articles reported RR data for circulating genistein (*n* = 9) [29,35,40,42,44,50,51,54,56], circulating daidzein (*n* = 7) [29,35,40,42,44,51,54], and total circulating isoflavones (*n* = 2) [29,54]. The pooled RR for circulating genistein and PCa risk was 0.87 (95% CI: 0.69–1.10, *p* = 0.236) (Figure 3A), the pooled RR for circulating daidzein and PCa risk was 0.92 (95% CI: 0.78–1.08) (Figure 3B), and the RR for circulating isoflavones and PCa risk was 1.01 (95% CI: 0.93–1.10, *p* = 0.738). None of the studies were significant for bias using Begg’s correlation test (*p* = 0.175, *p* = 0.368, and *p* = 1.00, respectively) or Egger’s linear regression test (*p* = 0.228, *p* = 0.197, and *p* = NA, respectively). The *I*^2^ index showed high heterogeneity amongst the studies included in the circulating genistein (76.8%) and circulating daidzein (58.1%) groups and low heterogeneity amongst the circulating isoflavone group (0.0%). Funnel plots used to explore publication bias are shown in Appendix A (Figure A2).

### 3.5. Subgroup Analysis

**Figure A3:**
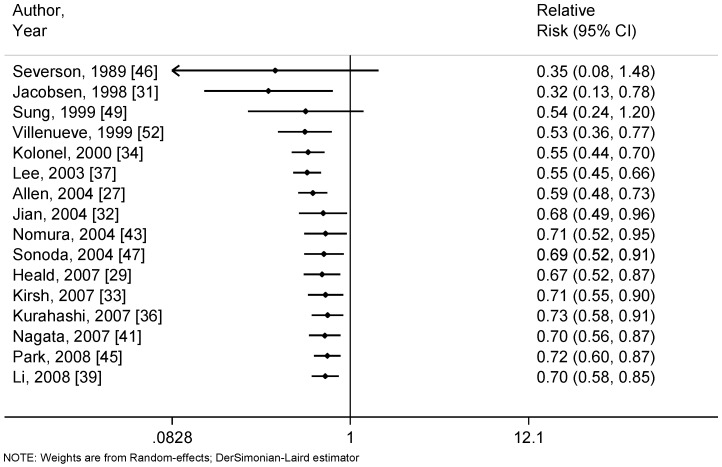
Forest plot for total soy intake and risk of prostate cancer by year of study publication. These associations were indicated as a relative risk (RR) estimate with the corresponding 95% confidence interval (CI).

Articles reporting total soy intake and PCa risk had a pooled RR of 0.61 (95% CI: 0.45–0.82, *p* = 0.001) for case-control studies and a pooled RR of 0.90 (95% CI: 0.82–0.99, *p* = 0.022) for cohort and NCC studies. Studies conducted in both North America (*p* = 0.009) and Europe (*p* = 0.021) were significantly associated with a reduced PCa risk, whereas studies conducted in Asia (*p* = 0.064) were not. A complete subgroup analysis can be found in Table 2. Cumulative meta-analyses first demonstrated that soy food intake was significantly associated with the reduced risk of PCa in 1998 and has remained significant over time, and with the inclusion of additional studies within the field (Figure A3).

Articles reporting unfermented soy food intake and PCa risk had a pooled RR of 0.55 (95% CI: 0.46–0.66, *p* < 0.001) for case-control studies and a pooled RR of 0.91 (95% CI: 0.76–1.08, *p* = 0.267) for cohort studies. Studies were conducted in both North America (*p* = 0.014) and Asia (*p* = 0.005), and there was a significantly reduced risk of PCa in both continents. Studies of medium quality were significantly associated with a lower PCa risk (*p* < 0.001). 

The pooled RR for articles reporting dietary genistein intake and PCa risk was 0.81 (95% CI: 0.68–0.96, *p* = 0.016) for case-control studies and a pooled RR of 0.93 (95% CI: 0.85–1.01, *p* = 0.077) for cohort and NCC studies. Studies conducted in Asia (*p* = 0.004) showed a significantly reduced risk of PCa, while studies conducted in North America (*p* = 0.145) and Europe (*p* = 0.419) did not. Mid quality studies were significantly associated with a reduced risk of PCa (*p* = 0.007). 

The pooled RR for articles reporting dietary daidzein intake and PCa risk was 0.68 (95% CI: 0.47–1.00, *p* = 0.052) for case-control studies and a pooled RR of 0.91 (95% CI: 0.84–1.00, *p* = 0.042) for cohort and NCC studies. Studies conducted in Asia (*p* = 0.012) showed a significantly reduced risk of PCa, while studies conducted in North America (*p* = 0.094) and Europe (*p* = 0.799) did not. High quality studies were significantly associated with a reduced risk of PCa (*p* = 0.042).

There was no significant association for case-control or NCC studies and risk of PCa for articles evaluating circulating levels of genistein. Only studies conducted in Asia (*p* = 0.031) were significantly associated with a decreased risk of PCa, while studies conducted in Europe (*p* = 0.784) showed no significant association with circulating genistein levels and PCa risk. 

Articles evaluating dietary isoflavones, fermented soy intake, and circulating levels of daidzein showed no significant associations on risk of PCa when studies were grouped by design or by continent. Finally, no studies significantly affected any of the pooled RRs when conducting sensitivity analyses for each group.

### 3.6. Soy and Advanced PCa Risk

**Figure 4:**
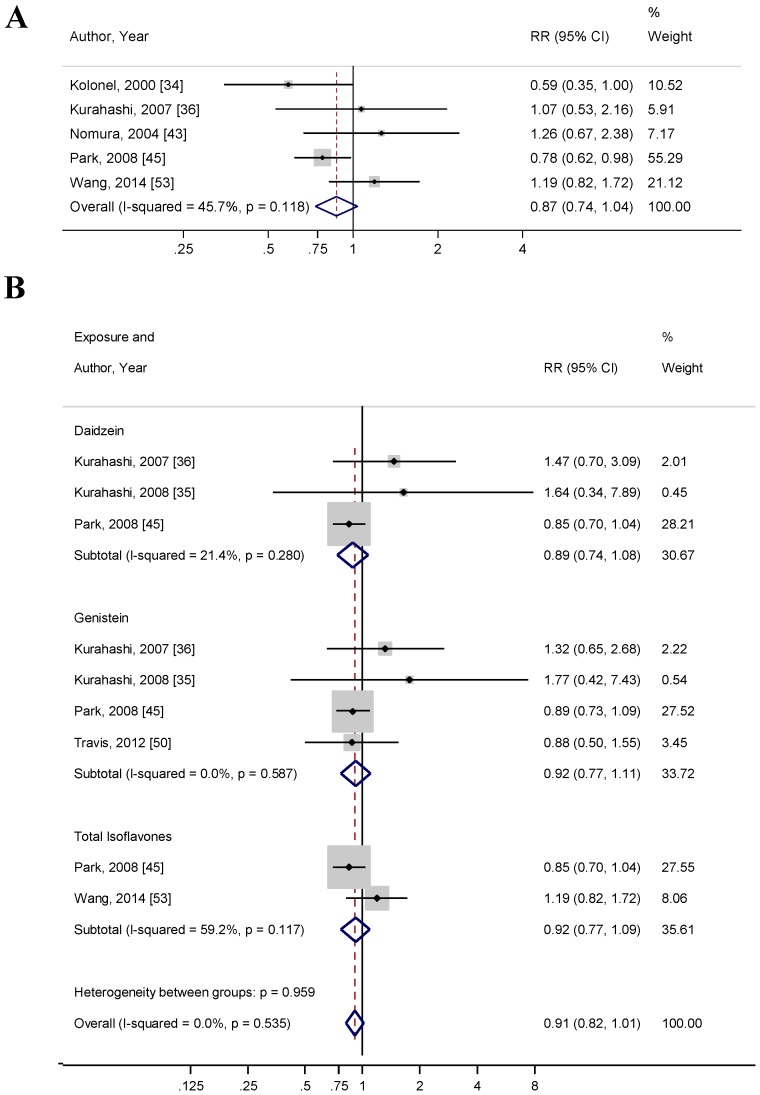
Forest plots for (**A**) total soy intake and risk of advanced prostate cancer; and (**B**) isoflavone exposure and risk of advanced prostate cancer. These associations were indicated as a relative risk (RR) estimate with the corresponding 95% confidence interval (CI).

**Figure A4:**
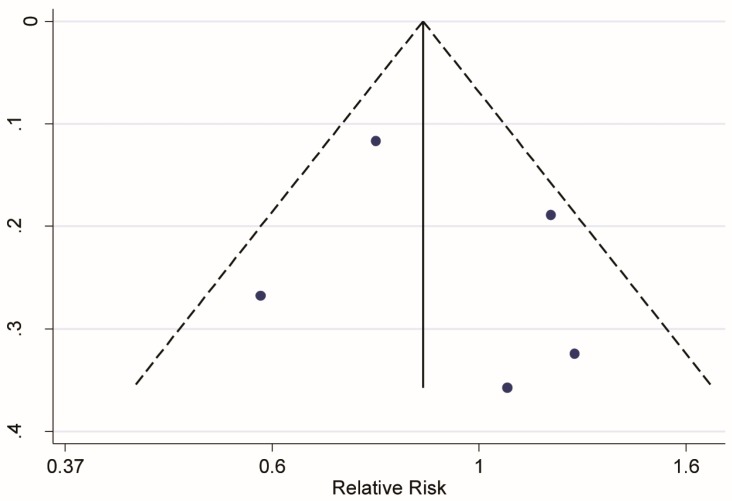
Funnel plot for total soy intake and risk of advanced prostate cancer.

Seven [34,35,36,43,45,50,53] studies reported the risk of advanced PCa with soy intake and circulating isoflavone levels. Of these seven studies, four [34,43,45,53] studies were conducted in North America, two [35,36] studies were conducted in Asia, and one [50] study was conducted in Europe. Only one [34] was a case-control study, two [35,50] were NCC studies, and four [36,43,45,53] were cohort studies. Five [34,36,43,45,53] studies reported a risk of advanced PCa with dietary soy food intake and two [35,50] studies reported a risk of advanced PCa with circulating isoflavones. For studies that reported dietary soy food intake, the pooled RR was 0.87 (95% CI: 0.74–1.06, *p* = 0.119) (Figure 4A). Neither Begg’s correlation test (*p* = 1.00) nor Egger’s linear regression test (*p* = 0.548) for bias were significant. The *I*^2^ index showed moderate heterogeneity between studies (45.7%). Funnel plots, used to explore publication bias, are shown in Appendix A (Figure A4).

When stratifying groups by combining dietary and circulating measurements of isoflavones, the pooled RR for genistein and PCa risk (*n* = 4) was 0.92 (95% CI: 0.77–1.11, *p* = 0.381), the pooled RR for daidzein and PCa risk (*n* = 3) was 0.89 (95% CI: 0.74–1.10, *p* = 0.227), and the pooled RR for total isoflavones and PCa risk (*n* = 2) was 0.91 (95% CI: 0.82–1.01, *p* = 0.337) (Figure 4B). The *I*^2^ index showed moderate heterogeneity amongst studies reporting genistein and daidzein dietary and circulating levels (33.7% and 30.7%, respectively), while there was substantially high heterogeneity between the studies reporting total isoflavone measurements (100.0%).

## 4. Discussion

This updated systematic review and meta-analysis provides a thorough evaluation regarding the association between soy food intake and PCa risk. Using the current pool of scientific literature, our results support the existing evidence, which indicates that total soy food intake is associated with a reduced risk of PCa (*p* < 0.001). This population-based evidence corroborates observations in both in vitro and in vivo studies, which have shown that soy isoflavones inhibit PCa development and growth [57,58,59]. In agreement with this, we found that both genistein and daidzein intake were inversely associated with the risk of PCa (*p* = 0.008 and *p* = 0.018, respectively). These results support our finding that total soy food consumption is associated with decreased PCa risk, as genistein and daidzein are likely found in similar food products. 

Soybeans and soy food products contain isoflavones—predominantly genistein and daidzein—mainly as β-glycosides [60]. During digestion, these glycosides are hydrolyzed to their aglycone forms by intestinal or bacterial β-glucosidases [60]. By removing the sugar molecule, the isoflavones are smaller and more hydrophobic, allowing them to more readily diffuse into enterocytes [61]. After absorption and first pass metabolism, aglycones are re-conjugated in the liver to their glycosidic or other conjugated forms and distributed to tissues via systemic circulation [61]. Once within cells, isoflavones act as weak estrogen receptor (ER) agonists or antagonists, depending on the cell type and concentration of estrogen present [61]. Prostatic tissues have higher concentrations of ER-β, to which genistein preferentially binds, with an affinity similar to that of the endogenously-produced estrogen, 17β-estradiol [62]. Increased presence and activation of ER-β is associated with reduced cell proliferation and reduced PCa histological grade [63,64]. This effect has been shown to occur, in part, by reducing the levels of prostate-specific antigen (PSA), cyclin D1, and cyclin-dependent kinase 4 (CDK4) in an ER-dependent manner [57,58]. Interestingly, ER-β expression is often lost during prostate carcinogenesis, so the ability of genistein to bind to ER-β may be a key factor in the inhibition of prostate carcinogenesis. Additional mechanisms of the effects of soy isoflavones on PCa cellular proliferation, apoptosis, and differentiation have been reviewed in depth by Mahmoud et al. (2014) [65]. 

We also analyzed the potential relationship between unfermented or fermented soy food products and risk of PCa. We found that unfermented soy food products were associated with a decreased risk of PCa (*p* < 0.001), while fermented soy food products had no associations with PCa risk (*p* = 0.281). More studies provided food intake data for unfermented soy food products than fermented soy food products (11 studies versus 8 studies, respectively). While this meta-analysis failed to demonstrate a significant association between fermented foods and the risk of PCa, it should be recognized that there was wider variation in results reported by these studies than there was for studies using unfermented and other soy foods. This wider variation could have impacted the risk outcomes. Some concerns have been expressed in the literature regarding the effects of soy fermentation on the risk of developing certain cancers, such as gastric cancer [66]. Due to this association, Yan and Spitznagel chose to not include fermented soy foods in their 2005 meta-analysis [10]. However, during fermentation, β-glucosidases, secreted by fermentative bacteria, cleave glycosidic linkages via a similar process that digestive enzymes in the small intestine and gut microbiota cleave these linkages [60,61,67]. Isoflavones are present in fermented foods, such as tempeh and miso, predominantly as aglycones, with few isoflavones retaining their side-chains. The ratio is reversed for nonfermented foods, but some naturally occurring plant β-glucosidase activity allows for continuous side-chain cleavage to yield aglycones [60]. The more bioavailable aglycone form is readily absorbed from the intestines, rendering this conversion from a glycosylated isoflavone to its aglycone counterpart essential for maximal isoflavone absorption. These similar enzymatic processes yield common aglycone products for intestinal absorption, regardless of whether these processes occur before digestion during the fermentation of soy foods or during the digestion of unfermented soy foods. 

Circulating levels of genistein and daidzein were not associated with the risk of PCa (*p* = 0.236 and *p* = 0.310, respectively) despite the elevation of circulating genistein and daidzein levels after consumption of these isoflavones. However, peak circulating isoflavone levels can occur as soon as 30 min or as long as 6 h after feeding, depending on the specific isoflavones and foods consumed [61,68]. Mean half-life values vary as well; the average half-life for free genistein is 3.2 h and the average half-life for free daidzein is 4.2 h [69]. Because isoflavones can exist with multiple side-chains (e.g., glucose, sulfate, acetyl, or malonyl-CoA groups) and as different metabolites, accurately measuring in vivo pharmacokinetics is challenging. Thus, the amount of time between isoflavone consumption and blood collection may substantially affect measurement outcomes, which can impact the reliability of blood isoflavone measurements as markers of soy food intake. Due to these factors, an association may have been missed for these circulating isoflavones.

No association was seen when we examined six articles that did not disclose the source of isoflavones measured from participants’ dietary intakes. For example, Bosetti et al. (2006) indicated that isoflavones were measured from the FFQ, primarily based on consumption of soy and soy products, but also from “vegetable or bean soups and pulses” [28]. Because isoflavones are found in other food sources and supplements, such as clover and alfalfa seeds and sprouts, garbanzo beans, and other pulses, we independently analyzed studies that did not explicitly indicate that measurements were taken from soy food sources. As such, total dietary isoflavones were not significantly associated with risk of PCa (*p* = 0.313). While most dietary isoflavones are consumed from soy food products, examining the sources and types of isoflavones that were included in these analyses could provide insight as to why no association was observed. Notably, this analysis was based on the information found from a limited number of articles, so additional studies could strengthen these observations. 

To our knowledge, this is the first meta-analysis to investigate the risk associations between soy and advanced PCa. Advanced PCa is defined as poorly differentiated, aggressive, and metastatic disease. Advanced PCa is often difficult to treat, as patients are typically less responsive to therapy. It is therefore imperative to identify other ways to prevent disease progression, such as through dietary modification. Relatively few studies have examined the relationship between diet and advanced PCa or reported information pertaining to stage or grade of PCa. Our results do not show a significant reduction in the risk of advanced PCa with total soy intake (*p* = 0.119) or dietary and circulating levels of genistein (*p* = 0.381), daidzein (*p* = 0.227), or total isoflavones (*p* = 0.337), perhaps due to the lack of studies. Two double-blinded, randomized, placebo-controlled clinical trials have supplemented isoflavones in men awaiting radical prostatectomy. One study reported higher apoptotic activity in tumors of men treated with isoflavones when compared to tumors of the men in the placebo group [70], while the other study showed modulation of both cell cycle and apoptotic genes in the prostate tissues of men in the treatment group, when compared to tissues of men in the placebo group [71]. More studies are needed to further explore this promising relationship and to identify whether soy can protect against advanced PCa.

Few studies have analyzed individual soy foods and their relationships with PCa risk. Tofu was the most investigated soy food found in the literature. Tofu showed a significant protective association with PCa (*p* = 0.013). This result is consistent with the result shown by Hwang et al. (2009) [11]. More studies are needed to understand the role of individual soy foods in PCa risk.

The research design of studies did not seem to bias our results, as case-control and cohort studies both reported significant and null results. It is important to note that case-control studies are generally considered to have a higher risk of bias than cohort studies; however, significant measurement error can occur for both cohort and case-control studies when evaluating a single exposure variable. To accurately account for all reported estimates of soy exposure and PCa risk, both study designs were included in the analysis. To this end, sensitivity analyses conducted for each subgroup showed that no studies significantly altered results or heterogeneity within subgroups. Separating results by continent showed that total soy intake was only associated with a decreased risk of PCa in North America (*p* = 0.009) and Europe (*p* = 0.021), although only one study was included in the European group. However, when looking at the individual dietary isoflavones, genistein and daidzein, Asia was the only continent to show a significant risk reduction for PCa (*p* = 0.004 and *p* = 0.012, respectively). Similarly, Asia was the only continent to show a significant association between circulating genistein and risk of PCa (*p* = 0.031). Both North America (*p* = 0.014) and Asia (*p* = 0.005) showed a reduced risk of PCa when only considering unfermented soy foods. The variability in these associations makes it difficult to draw conclusions about whether ethnic differences, preparation methods, or eating patterns exist in soy food or isoflavone consumption and PCa risk. 

Because differences in overall dietary composition or other unmeasured lifestyle factors could contribute to increased or decreased disease risk, the results from this study and others showing that soy intake is associated with a reduced risk of PCa should be interpreted with caution. Our study was limited in that our results relied on the reporting of the studies included in this analysis and may have been affected by several factors. For example, studies relying on dietary recall or FFQ reporting are subject to recall bias by participants. Soy food intake measurements could be inconsistently reported or nutrient analyses may differ based on the amount and type of soy food or the database used to collect nutrient information. In addition, not all studies accounted for potential confounding variables, such as family history of prostate cancer (FHPC), body mass index (BMI), smoking, or energy intake. The subgroup analyses in this study attempted to account for these limitations by highlighting some of these differences between studies, to account for variability in data adjustments and selection bias created by study design (i.e., whether the study was case-control or NCC/cohort).

In addition to attempting to address these study limitations, our analysis delved deeper into potential confounding factors, through meta-regression, to determine whether study quality, length, or the sample size impacted significant results of the study. None of these factors were found to impact our results, and as such were not included in our results. We also reported both dietary intakes and circulating levels of isoflavones to create a more comprehensive review of the existing literature. Finally, we analyzed any associations between soy and advanced PCa, which has not previously been reported.

As the second most commonly diagnosed cancer in men worldwide, it is important to identify modifiable factors, such as diet, that may impact the risk of developing PCa. The current study provides an updated systematic review and meta-analysis of the available literature describing the associations between soy food consumption and PCa risk. Of the four meta-analyses previously published, all showed that soy intake was associated with a reduced risk for PCa. Our study further enhances this association by including additional studies for analysis, grouping soy foods by type of food and by isoflavone intake, adding groups of circulating isoflavone concentrations, and by evaluating the potential relationship between soy food intake and advanced PCa risk. 

## Figures and Tables

**Table 1:** Characteristics of included studies.

Author, Year	Country	Exposure	Exposure Type	Cases/Sample	Study Type	Study Period (Years)	Adjustments	Participant Age Range, Mean (SD)	Exposure Measurement (Intake or Circulating)
Allen, 2004 [27]	Japan	Total soy, miso, tofu	Diet	196/18,115	Cohort	1950–1996	Age, education, geographic area, city of residence, radiation dose	51–89 Cases: 75	Tofu, miso: T1 < 2x/week, T3 = almost daily Total soy: T1 = low, T3 = high
Bosetti, 2006 [28]	Italy	Total isoflavones	Diet	1294/2745	Case-Control	1991–2002	Age, BMI, FHPC, education, energy, study center	46–74 Cases: 66 Controls: 63	Q1 ≤ 14.7 μg/day, Q5 > 32.2 μg/day
Heald, 2007 [29]	Scotland	Total isoflavones, total soy	Diet	437/920	Case-Control	1998–2001	Age, smoking, FHPC, energy, family history of breast cancer, Carstairs Deprivation Index, smoking and energy intake:BMR ratio	50–74 Cases: 67.2 (5.5) Controls: 66.0 (5.4)	Isoflavones (μg/day): Q1 < 581.1, Q4 > 1982.2 Soy food: no, yes
		Genistein, daidzein, total isoflavones	Serum				Age, smoking, FHPC, family history of breast cancer, Carstairs Deprivation Index		Genistein (nmol/L): Q1 < 14.23, Q4 > 64.53 Daidzein (nmol/L): Q1 < 8.26, Q4 > 29.11 Isoflavones (nmol/L): Q1 < 25.57, Q4 > 98.86
Hedelin, 2006 [30]	Sweden	Total isoflavones, genistein, daidzein	Diet	1499/2629	Case-Control	2001–2002	Age, energy	35–79 Cases: 66.8 Controls: 67.8	Total isoflavones (μg/day): Q1 ≤ 1.0, Q4 ≥ 2.6 Genistein (μg/day): Q1 ≤ 0.27, Q4 ≥ 1.08 Daidzein (μg/day): Q1 ≤ 0.49, Q4 ≥ 1.11
Jacobsen, 1998 [31]	USA	Soy milk	Diet	225/12,395	Cohort	1976–1992	Age	≥25	Never, <daily, 1x/day, >1x/day
Jian, 2004 [32]	China	Total soy	Diet	130/404	Case-Control	2001–2002	Age, BMI, FHPC, education, PA, energy, geographic area, marital status, income, fresh vegetables and fruit consumption, tea drinking	Cases: 74.7 (7.1) Controls: 71.4 (7.2)	T1 = 0 g/day, T3 > 4.00 g/day
Kirsh, 2007 [33]	USA	Total soy	Diet	1338/29,361	Cohort	1993–2001	Age, BMI, smoking, FHPC, PA, energy, ethnicity, geographic area, supplemental vitamin E, total fat intake, red meat intake, diabetes, aspirin use, previous number of PCa screening examinations	63.3	Q1 = 0 servings/month, Q4 > 0.5 servings/month
Kolonel, 2000 [34]	USA, Canada	Total soy	Diet	1619/3237	Case-Control	1987–1991	Age, education, energy, ethnicity, geographic area	≥65	Q1 < 0.1 g/day, Q5 > 39.4 g/day
Kurahashi, 2007 [36]	Japan	Total soy, miso, genistein, daidzein	Diet	307/43,509	Cohort	1995–2004	Age, geographic area	45–74	Total soy (g/day): Q1 < 46.6, Q4 ≥ 107.4 Miso (mL/day): Q1 < 110.0, Q4 ≥ 356.0 Genistein (mg/day): Q1 < 13.2, Q4 ≥ 32.8 Daidzein (mg/day): Q1 < 8.5, Q4 ≥ 20.4
Kurahashi, 2008 [35]	Japan	Genistein, daidzein	Plasma	201/603	Nested Case-Control	1990–2005	Smoking; alcohol; marital status; intake of green tea, protein, fiber, green or yellow vegetables	40–69 Cases: 58.6 (6.4) Controls: 58.4 (6.6)	Genistein (ng/mL): T1 < 57, T3 ≥ 151.7 Daidzein (ng/mL): T1 < 22, T3 ≥ 61.5
Lee, 2003 [37]	China	Total soy, tofu, genistein, daidzein	Diet	133/398	Case-Control	1989–1992	Age, energy	50–89	Total soy (g/day): Q1 < 27.5, Q4 > 111.8 Tofu (g/day): T1 < 14.3, T3 > 34.5 Genistein (mg/day): Q1 < 17.9, Q4 > 62.0 Daidzein (mg/day): Q1 < 10.0, Q4 > 36.3
Lewis, 2009 [38]	USA	Genistein, daidzein	Diet	478/860	Case-Control	1998–2004	Age, BMI, smoking, FHPC, education, energy	Controls: 62.0 (10.7) Incident cases: 63.3 (8.2) Prevalent cases: 66.9 (8.1)	Genistein (mcg/day): L ≤ 196.0, U > 196.1 Daidzein (mcg/day): L ≤ 77.0, U > 77.1
Li, 2008 [39]	China	Total soy	Diet	28/308	Case-Control	1998–2000	BMI, smoking, education, alcohol, food frequency	Cases: 71.39 (6.03) Control: 71.14 (5.78)	T1 ≤ 2x/week T3 ≥ 1x/day
Low, 2006 [40]	Europe	Genistein, daidzein	Diet	85/241	Nested Case-Control	1993–1997	BMI, FHPC, energy	45–75	Genistein average (95% CI) cases (μg/day): 287.7 (255.5–323.9); controls: 310.2 (283.0–339.9) Daidzein average (95% CI) cases (μg/day): 224.4 (198.1–254.2); controls: 249.2 (227.8–272.5)
			Plasma						Genistein average (95% CI) cases (ng/mL): 4.8 (3.6–6.4); controls: 4.4 (3.7–5.4) Daidzein average (95% CI) cases (ng/mL): 2.4 (1.8–3.1); controls: 2.4 (2.0–2.9)
Nagata, 2007 [41]	Japan	Total soy, genistein, daidzein	Diet	200/400	Case-Control	1996–2003	Smoking, energy	59–73	Total soy (isoflavones) (mg/day): Q1 < 30.5, Q4 ≥ 89.9 Genistein (mg/day): Q1 < 1.1, Q4 ≥ 2.5 Daidzein (mg/day): Q1 < 0.8, Q4 ≥ 1.9
Nagata, 2016 [42]	Japan	Genistein, daidzein	Diet	56/112	Case-Control	2011–2014	Age, BMI, smoking, alcohol, energy	Cases: 64.7 (6.6) Controls: 63.6 (9.1)	Genistein (mg/day): T1 < 17.57, T3 ≥ 36.31 Daidzein (mg/day): T1 < 11.56, T3 ≥ 21.86
		Genistein, daidzein	Serum				Age, BMI, smoking, alcohol		Genistein (ng/mL): T1 < 57.10, T3 ≥ 144.50 Daidzein (ng/mL): T1 < 18, T3 ≥ 51.7
Nomura, 2004 [43]	USA	Tofu	Diet	222/5826	Cohort	1971–1995	Age, BMI, smoking, alcohol, energy, arm muscle area	Not given	Q1 = 0 g/week, Q5 > 240 g/week
Ozasa, 2004 [44]	Japan	Genistein, daidzein	Serum	52/203	Nested Case-Control	1988–1999	Age	≥40 Cases: 69.4 Controls: 68.7	Genistein (nM): T1 < 239, T3 > 682 Daidzein (nM): T1 < 89, T3 > 239
Park, 2008 [45]	USA	Total soy, total isoflavones, genistein, daidzein	Diet	4404/82,483	Cohort	1993–1996	Time since cohort entry, ethnicity, FHPC, education, BMI, smoking, energy	45–75	Total soy (g/1000 kcal): T1: 0, T2: 0.1–2.8, T3: ≥2.8 Genistein (mg/1000 kcal): Q1 < 0.7, Q2: 0.7–1.2, Q3: 1.2–1.9, Q4: 1.9–3.1, Q5 ≥ 3.1 Daidzein (mg/1000 kcal): Q1 < 0.7, Q2: 0.7–1.3, Q3: 1.3–2.0, Q4: 2.0–3.2, Q5 ≥ 3.2 Total isoflavones (mg/1000 kcal): Q1 < 1.6, Q2: 1.6–2.9, Q3: 2.9–4.5, Q4: 4.5–7.2, Q5 ≥ 7.2
Severson, 1989 [46]	USA	Miso, tofu	Diet	174/7999	Cohort	1965–1986	Age	≥46	Miso: T1 ≤ 1x/week, T3 ≥ 5x/week Tofu: T1 ≤ 1x/week, T3 ≥ 5x/week
Sonoda, 2004 [47]	Japan	Total soy, natto, tofu	Diet	140/280	Case-Control	1996–2002	Smoking, energy	59–73	Total soy (g/day): Q1 ≤ 77.0, Q4 ≥ 187.2 Tofu (g/day): Q1 ≤ 19.7, Q4 ≥ 96.4 Natto (g/day): Q1 ≤ 5.7, Q4 ≥ 40.0
Strom, 1999 [48]	USA	Genistein, daidzein	Diet	83/190	Case-Control	1996–1998	Age, FHPC, alcohol, energy	Cases: 61 (6.6) Controls: 60.6 (6.9)	Genistein mean (μg/day): cases: 19.8; controls: 29.7 Daidzein mean (μg/day): cases: 14.2; controls: 22.8
Sung, 1999 [49]	China	Soy milk	Diet	90/270	Case-Control	1995–1996	None	≥50	Yes, No
Travis, 2009 [51]	Europe	Genistein, daidzein	Plasma	950/1992	Nested Case-Control	1992–2003	BMI, smoking, education, PA, alcohol, marital status	43–76 Cases: 60.4 (5.8) Controls: 60.1 (5.8)	Genistein (ng/mL): Q1 ≤ 0.30, Q5 ≥ 7.00 Daidzein (ng/mL): Q1 ≤ 0.30, Q5 ≥ 4.10
Travis, 2012 [50]	Europe	Genistein	Plasma	655/1310	Nested Case-Control	1992–2006	BMI, smoking, education, PA, alcohol, marital status	43–76 Cases: 60.4 (5.8) Controls: 60.1 (5.8)	Genistein (ng/mL): Q1 ≤ 0.30, Q5 ≥ 6.10
Villenueve, 1999 [52]	Canada	Total soy	Diet	1623/3246	Case-Control	1994–1997	Age, geographic area	50–74	None, some
Wang, 2014 [53]	USA	Total isoflavones	Diet	3974/43,268	Cohort	1999–2009	Age	50–74	Q1 < 0.029 mg/day, Q5 ≥ 0.144 mg/day
Ward, 2008 [54]	Europe	Total isoflavones, genistein, daidzein	Plasma	194/1006	Nested Case-Control	1993–2006	Age, energy	40–79	Total isoflavones median (ng/mL): 10.3 Genistein median (ng/mL): 6.9 Daidzein median (ng/mL): 2.5
Ward, 2010 [55]	Europe	Total isoflavones, genistein, daidzein	Diet	204/1016	Nested Case-Control	1993–2006	Age	40–79	Total isoflavones mean cases (μg/day): 948.6; controls: 1088 Genistein: mean cases (μg/day): 546.6; controls: 638.2 Daidzein: mean cases (μg/day): 314.9; controls: 355.2
Wu, 2015 [56]	China	Genistein	Plasma	46/100	Case-Control	2012–2013	Age	70.1 (8.9) Cases: 72.5 (8.4) Control: 68.0 (8.8)	<640.2 nmol/L, >640.0 nmol/L

Abbreviations: body mass index (BMI), family history of prostate cancer (FHPC), basal metabolic rate (BMR), physical activity (PA).

**Table 2:** Subgroup analysis of included studies. Bold values indicate *p* < 0.05.

	**Total Dietary Soy**				**Dietary Unfermented Soy**				**Dietary Fermented Soy**			
	**No. of Studies**	**RR (95% CI)**	***p*-Value**	***I*^2^ (%)**	**No. of Studies**	**RR (95% CI)**	***p*-Value**	***I*^2^ (%)**	**No. of Studies**	**RR (95% CI)**	***p*-Value**	***I*^2^ (%)**
**Overall Model**	16	0.71 (0.58–0.85) ^†^	**<0.001**	68.9	11	0.66 (0.52–0.83) ^†^	**<0.001**	60.3	8	0.86 (0.66–1.13) ^†^	0.281	66.6
**Study Type**												
**Case-control**	9	0.61 (0.45–0.82) ^†^	**0.001**	63.2	6	0.55 (0.46–0.66)	**<0.001**	0.0	4	0.64 (0.27–1.50) ^†^	0.300	82.3
**Cohort**	7	0.90 (0.82–0.99)	**0.022**	2.7	5	0.91 (0.76–1.08)	0.267	30.6	4	0.92 (0.83–1.02)	0.123	0.0
**Continent**												
**North America**	7	0.72 (0.56–0.92) ^†^	**0.009**	74.7	6	0.65 (0.47–0.92) ^†^	**0.014**	73.4	2	0.91 (0.81–1.02)	0.090	0.0
**Europe**	1	0.52 (0.30–0.91)	**0.021**	0.0		**-**	**-**	**-**	**-**	**-**	**-**	**-**
**Asia**	8	0.71 (0.50–1.02) ^†^	0.064	67.3	5	0.68 (0.52–0.89)	**0.005**	35.4	6	0.79 (0.51–1.23) ^†^	0.302	75.5
**Adjustments**												
**High quality**	12	0.72 (0.58–0.90) ^†^	**0.003**	73.4	8	0.61 (0.45–0.82) ^†^	**0.001**	69.2	5	1.00 (0.73–1.37) ^†^	0.994	63.1
**Mid quality**	4	0.66 (0.50–0.87)	**0.003**	60.5	3	0.81 (0.58–1.13)	0.217	0.0	3	0.56 (0.27–1.17) ^†^	0.124	74.0
**Age**												
**Adjusted**	11	0.73 (0.57–0.93) ^†^	**0.010**	69.7	8	0.68 (0.52–0.88) ^†^	**0.003**	65.6	5	1.03 (0.72–1.47) ^†^	0.892	59.8
**Unadjusted**	5	0.61 (0.39–0.96) ^†^	**0.032**	32.1	3	0.56 (0.35–0.92)	**0.021**	47.5	3	0.56 (0.28–1.15) ^†^	0.115	0.0
**BMI**												
**Adjusted**	5	0.95 (0.73–1.22) ^†^	0.661	66.2	3	0.78 (0.50–1.23) ^†^	0.282	64.6	2	1.27 (0.58–2.78) ^†^	0.552	83.8
**Unadjusted**	11	0.61 (0.53–0.71)	**<0.001**	32.0	8	0.59 (0.50–0.70)	**<0.001**	18.0	6	0.73 (0.50–1.07) ^†^	0.103	63.3
**Smoking**												
**Adjusted**	7	0.71 (0.56–0.91) ^†^	**0.007**	66.3	4	0.71 (0.46–1.10) ^†^	0.122	62.2	3	0.56 (0.28–1.15) ^†^	0.115	78.7
**Unadjusted**	9	0.72 (0.53–0.96) ^†^	**0.027**	66.9	7	0.60 (0.51–0.71)	**<0.001**	27.1	5	1.03 (0.72–1.47) ^†^	0.892	59.8
**FHPC**												
**Adjusted**	4	0.95 (0.72–1.24) ^†^	0.690	71.6	1	0.98 (0.79–1.22)	0.855	0.0	2	1.27 (0.58–2.78) ^†^	0.552	83.8
**Unadjusted**	12	0.63 (0.55–0.72)	**<0.001**	41.5	10	0.61 (0.52–0.72)	**<0.001**	32.0	5	0.73 (0.50–1.07) ^†^	0.103	63.3
**Energy**												
**Adjusted**	9	0.76 (0.61–0.95) ^†^	**0.016**	69.7	5	0.74 (0.57–0.97) ^†^	**0.029**	53.2	5	0.72 (0.43–1.22) ^†^	0.221	79.2
**Unadjusted**	7	0.62 (0.45–0.86) ^†^	**0.004**	57.1	6	0.57 (0.39–0.83) ^†^	**0.004**	50.6	3	1.01 (0.80–1.28)	0.919	0.0
**Education**												
**Adjusted**	5	0.82 (0.58–1.16) ^†^	0.263	74.7	3	0.64 (0.41–0.99) ^†^	**0.045**	56.1	3	1.06 (0.76–1.47) ^†^	0.741	67.7
**Unadjusted**	11	0.64 (0.50–0.82) ^†^	**<0.001**	63.4	8	0.66 (0.48–0.89) ^†^	**0.007**	65.5	5	0.66 (0.39–1.11) ^†^	0.116	68.2
**PA**												
**Adjusted**	2	1.32 (0.66–2.66) ^†^	0.433	78.1	1	0.98 (0.79–1.22)	0.855	0.0	1	2.02 (1.08–3.78)	**0.028**	0.0
**Unadjusted**	14	0.64 (0.52–0.78) ^†^	**<0.001**	63.6	10	0.61 (0.52–0.72)	**<0.001**	32.0	7	0.79 (0.62–1.02) ^†^	0.071	58.1
**Alcohol**												
**Adjusted**	2	0.55 (0.19–1.59) ^†^	0.270	74.4	2	0.55 (0.19–1.59) ^†^	0.270	74.7				
**Unadjusted**	14	0.71 (0.58–0.87) ^†^	**0.001**	70.4	9	0.66 (0.51–0.85) ^†^	**0.002**	62.3	8	0.86 (0.66–1.13) ^†^	0.281	66.6
**Geographic area**												
**Adjusted**	6	0.83 (0.61–1.13) ^†^	0.237	80.1	4	0.72 (0.51–1.02) ^†^	0.064	80.7	3	1.16 (0.81–1.67) ^†^	0.416	55.2
**Unadjusted**	10	0.59 (0.45–0.79) ^†^	**<0.001**	60.9	7	0.63 (0.48–0.82)	**0.001**	28.1	5	0.64 (0.41–1.02) ^†^	0.061	68.9
**Marital status**												
**Adjusted**	1	2.02 (1.08–3.78)	**0.028**	0.0	**-**	**-**	**-**	**-**	1	2.02 (1.08–3.78)	**0.028**	0.0
**Unadjusted**	15	0.67 (0.56–0.81) ^†^	**<0.001**	58.5	11	0.66 (0.52–0.83) ^†^	**<0.001**	60.3	7	0.79 (0.62–1.02) ^†^	0.071	58.1
**Ethnicity**												
**Adjusted**	3	0.86 (0.71–1.04) ^†^	0.124	58.6	2	0.80 (0.51–1.25) ^†^	0.319	78.7	**-**	**-**	**-**	**-**
**Unadjusted**	13	0.65 (0.50–0.84) ^†^	**0.001**	62.6	9	0.61 (0.51–0.73)	**<0.001**	39.5	8	0.86 (0.66–1.13) ^†^	0.281	66.6
	**Dietary Isoflavones**				**Dietary Genistein**				**Dietary Daidzein**			
	**No. of studies**	**RR (95% CI)**	***p*-Value**	***I*^2^ (%)**	**No. of studies**	**RR (95% CI)**	***p*-Value**	***I*^2^ (%)**	**No. of studies**	**RR (95% CI)**	***p*-Value**	***I*^2^ (%)**
**Overall Model**	6	1.03 (0.97–1.09)	0.313	44.9	10	0.90 (0.84–0.97)	**0.008**	31.0	10	0.84 (0.73–0.97) ^†^	**0.018**	50.5
**Study Type**												
**Case-control**	3	1.04 (0.89–1.22)	0.604	0.0	6	0.81 (0.68–0.96)	**0.016**	49.5	6	0.68 (0.47–1.00) ^†^	0.052	70.1
**Cohort/NCC**	3	1.01 (0.87–1.17) ^†^	0.928	76.3	4	0.93 (0.85–1.01)	0.077	0.0	4	0.91 (0.84–1.00)	**0.042**	0.0
**Continent**												
**North America**	2	1.03 (0.85–1.26)	0.757	86.2	3	0.76 (0.53–1.10) ^†^	0.145	62.0	3	0.71 (0.47–1.06) ^†^	0.094	68.2
**Europe**	4	1.00 (0.89–1.12)	0.961	0.0	3	0.95 (0.84–1.08)	0.419	0.0	3	0.98 (0.80–1.18) ^†^	0.799	51.7
**Asia**	**-**	**-**	**-**	**-**	4	0.69 (0.53–0.89)	**0.004**	0.0	4	0.72 (0.55–0.93)	**0.012**	0.0
**Adjustments**												
**High quality**	5	1.02 (0.91–1.14) ^†^	0.780	55.8	8	0.90 (0.83–0.98)	**0.011**	25.1	8	0.88 (0.81–0.96)	**0.004**	28.1
**Mid quality**	1	1.05 (0.84–1.31)	0.667	0.0	2	0.81 (0.47–1.38) ^†^	0.430	72.5	2	0.83 (0.40–1.72) ^†^	0.620	84.4
**Age**												
**Adjusted**	5	1.08 (1.00–1.16)	**0.042**	8.1	7	0.89 (0.79–1.00)	**0.046**	36.6	7	0.83 (0.66–1.03) ^†^	0.092	56.7
**Unadjusted**	1	0.93 (0.83–1.04)	0.207	0.0	3	0.91 (0.83–1.01)	0.077	42.4	3	0.81 (0.64–1.03) ^†^	0.088	51.0
**BMI**												
**Adjusted**	2	0.94 (0.85–1.04)	0.224	0.0	4	0.91 (0.82–1.00)	0.058	39.0	4	0.89 (0.80–0.98)	**0.018**	38.7
**Unadjusted**	4	1.09 (1.01–1.18)	**0.029**	19.6	6	0.90 (0.80–1.01)	0.063	38.2	6	0.83 (0.66–1.05) ^†^	0.116	61.2
**Smoking**												
**Adjusted**	2	0.95 (0.85–1.06)	0.322	21.5	4	0.73 (0.51–1.04) ^†^	0.080	59.6	4	0.71 (0.50–1.01) ^†^	0.059	59.4
**Unadjusted**	4	1.08 (1.00–1.16)	0.055	27.8	6	0.91 (0.81–1.01)	0.086	10.8	6	0.88 (0.73–1.06) ^†^	0.178	51.1
**FHPC**												
**Adjusted**	3	0.95 (0.86–1.05)	0.331	0.0	4	0.90 (0.82–1.00)	**0.043**	45.7	4	0.76 (0.59–0.98) ^†^	**0.032**	55.9
**Unadjusted**	3	1.09 (1.00–1.18)	**0.040**	44.0	6	0.90 (0.80–1.02)	0.089	33.5	6	0.88 (0.71–1.09) ^†^	0.228	52.0
**Energy**												
**Adjusted**	4	0.97 (0.88–1.06)	0.477	0.0	8	0.90 (0.82–0.98)	**0.015**	42.7	8	0.78 (0.63–0.96) ^†^	**0.017**	60.6
**Unadjusted**	2	1.05 (0.87–1.27) ^†^	0.600	71.2	2	0.92 (0.79–1.07)	0.283	0.0	2	0.93 (0.80–1.09)	0.393	0.0
**Education**												
**Adjusted**	2	0.94 (0.85–1.04)	0.224	0.0	2	0.75 (0.44–1.28) ^†^	0.296	78.2	2	0.75 (0.45–1.24) ^†^	0.261	76.3
**Unadjusted**	4	1.09 (1.01–1.18)	**0.029**	19.6	8	0.89 (0.80–0.99)	**0.033**	15.5	8	0.91 (0.82–1.02)	0.104	49.8
**PA**												
**Adjusted**	**-**	**-**	**-**	**-**	**-**	**-**	**-**	**-**	**-**	**-**	**-**	**-**
**Unadjusted**	6	1.03 (0.97–1.10)	0.313	44.9	10	0.90 (0.84–0.97)	**0.008**	31.0	10	0.84 (0.73–0.97) ^†^	**0.018**	50.5
**Alcohol**												
**Adjusted**	**-**	**-**	**-**	**-**	2	0.74 (0.44–1.25)	0.255	0.0	2	0.62 (0.36–1.05)	0.075	0.0
**Unadjusted**	6	1.03 (0.97–1.10)	0.313	44.9	8	0.91 (0.84–0.98)	**0.012**	43.5	8	0.86 (0.74–0.99) ^†^	**0.042**	56.0
**Geographic area**												
**Adjusted**	**-**	**-**	**-**	**-**	1	0.80 (0.56–1.14)	0.219	0.0	1	0.87 (0.61–1.25)	0.447	0.0
**Unadjusted**	6	1.03 (0.97–1.10)	0.313	44.9	9	0.91 (0.84–0.98)	**0.015**	36.4	9	0.83 (0.71–0.97) ^†^	**0.022**	55.9
**Marital status**												
**Adjusted**	**-**	**-**	**-**	**-**	**-**	**-**	**-**	**-**	**-**	**-**	**-**	**-**
**Unadjusted**	6	1.03 (0.97–1.10)	0.313	44.9	10	0.90 (0.84–0.97)	**0.008**	31.0	10	0.84 (0.73–0.97) ^†^	**0.018**	50.5
**Ethnicity**												
**Adjusted**	1	0.93 (0.83–1.04)	0.207	0.0	1	0.94 (0.85–1.05)	0.256	0.0	1	0.92 (0.83–1.03)	0.134	0.0
**Unadjusted**	5	1.08 (1.00–1.16)	**0.042**	8.1	9	0.87 (0.78–0.97)	**0.009**	33.2	9	0.79 (0.65–0.96) ^†^	**0.019**	55.6
**Circulating Genistein**					**Circulating Daidzein**							
	**No. of studies**	**RR (95% CI)**	***p*-Value**	***I*^2^ (%)**	**No. of studies**	**RR (95% CI)**	***p*-Value**	***I*^2^ (%)**				
**Overall Model**	9	0.87 (0.69–1.10) ^†^	0.236	76.8	7	0.92 (0.78–1.08) ^†^	0.310	58.1				
**Study Type**												
**Case-control**	3	0.31 (0.06–1.65) ^†^	0.170	90.4	2	0.49 (0.06–3.92) ^†^	0.502	90.9				
**Cohort**	**-**	**-**	**-**	**-**	**-**	**-**	**-**	**-**				
**Nested Case-control**	6	0.97 (0.83–1.13) ^†^	0.668	52.0	5	0.98 (0.93–1.04)	0.490	0.0				
**Continent**												
**North America**	**-**	**-**	**-**	**-**	**-**	**-**	**-**	**-**				
**Europe**	5	1.02 (0.87–1.21) ^†^	0.784	58.7	4	0.99 (0.93–1.04)	0.657	6.8				
**Asia**	4	0.37 (0.15–0.92) ^†^	**0.031**	79.1	3	0.52 (0.22–1.23) ^†^	0.137	72.5				
**Adjustments**												
**High quality**	9	0.87 (0.69–1.10) ^†^	0.236	76.8	7	0.92 (0.78–1.08) ^†^	0.310	58.1				
**Mid quality**	**-**	**-**	**-**	**-**	**-**	**-**	**-**	**-**				
**Age**												
**Adjusted**	5	0.41 (0.13–1.29) ^†^	0.128	85.6	4	0.77 (0.44–1.35) ^†^	0.362	74.3				
**Unadjusted**	4	0.97 (0.72–1.29) ^†^	0.814	69.9	3	0.95 (0.83–1.08)	0.397	13.1				
**BMI**												
**Adjusted**	4	0.78 (0.45–1.34) ^†^	0.368	87.6	3	0.72 (0.44–1.16) ^†^	0.177	82.7				
**Unadjusted**	5	0.84 (0.60–1.17) ^†^	0.309	60.5	4	0.99 (0.93–1.05)	0.706	0.0				
**Smoking**												
**Adjusted**	4	0.73 (0.34–1.57) ^†^	0.424	88.2	3	0.66 (0.30–1.47) ^†^	0.307	81.9				
**Unadjusted**	5	0.90 (0.74–1.10) ^†^	0.272	55.6	4	0.99 (0.93–1.05)	0.682	0.0				
**FHPC**												
**Adjusted**	2	1.04 (0.86–1.26)	0.670	0.00	2	1.03 (0.89–1.2)	0.699	0.0				
**Unadjusted**	7	0.73 (0.51–1.05) ^†^	0.087	81.9	5	0.77 (0.58–1.06) ^†^	0.115	69.1				
**Energy**												
**Adjusted**	2	0.99 (0.94–1.05)	0.779	0.0	2	0.99 (0.94–1.05)	0.796	0.0				
**Unadjusted**	7	0.69 (0.42–1.13) ^†^	0.140	81.4	5	0.76 (0.50–1.15) ^†^	0.195	64.0				
**Education**												
**Adjusted**	2	1.07 (0.51–2.23) ^†^	0.866	88.2	1	0.80 (0.60–1.07)	0.131	0.0				
**Unadjusted**	7	0.80 (0.60–1.07) ^†^	0.126	76.8	6	0.94 (0.78–1.14) ^†^	0.528	59.6				
**PA**												
**Adjusted**	2	1.07 (0.51–2.23) ^†^	0.866	88.2	1	0.80 (0.60–1.07)	0.131	0.0				
**Unadjusted**	7	0.80 (0.60–1.07) ^†^	0.126	76.8	6	0.94 (0.78–1.14) ^†^	0.528	59.6				
**Alcohol**												
**Adjusted**	3	0.55 (0.20–1.51) ^†^	0.244	91.6	2	0.39 (0.08–1.88) ^†^	0.243	86.5				
**Unadjusted**	6	0.93 (0.77–1.12) ^†^	0.437	51.0	5	0.99 (0.94–1.05)	0.757	0.0				
**Geographic area**												
**Adjusted**	**-**	**-**	**-**	**-**	**-**	**-**	**-**	**-**				
**Unadjusted**	9	0.87 (0.69–1.10) ^†^	0.236	76.8	7	0.92 (0.78–1.08) ^†^	0.310	58.1				
**Marital status**												
**Adjusted**	2	1.07 (0.51–2.23) ^†^	0.866	88.2	1	0.80 (0.60–1.07)	0.131	0.0				
**Unadjusted**	7	0.80 (0.60–1.07) ^†^	0.126	76.8	6	0.94 (0.78–1.14) ^†^	0.528	59.6				
**Ethnicity**												
**Adjusted**	**-**	**-**	**-**	**-**	**-**	**-**	**-**	**-**				
**Unadjusted**	9	0.87 (0.69–1.10) ^†^	0.236	76.8	7	0.92 (0.78–1.08) ^†^	0.310	58.1				

^†^ Signifies that results are estimated by DerSimonian–Laird random effects model. Abbreviations: relative risk (RR), confidence interval (CI), number (No.), body mass index (BMI), family history of prostate cancer (FHPC), physical activity (PA).

## Appendix A

**Table A1:** Quality assessment of included studies.

Source	Selection				Comparability ^5^	Exposure			Total ^9^
Author, Year	Definition ^1^	Representative ^2^	Selection ^3^	Definition ^4^		Ascertainment ^6^	Method ^7^	Rate ^8^	
Allen, 2004 [27]	0	★	0	★	★	★	★	★	6
Bosetti, 2006 [28]	★	★	0	★	★★	0	★	★	7
Heald, 2007 [29]	★	★	★	★	★★	★	★	0	8
Hedelin, 2006 [30]	★	★	★	★	★	0	★	0	6
Jacobsen, 1998 [31]	★	★	0	★	★	★	★	★	7
Jian, 2004 [32]	★	★	0	★	★★	0	★	★	7
Kirsh, 2007 [33]	★	★	0	★	★★	★	★	★	8
Kolonel, 2000 [34]	★	★	★	★	★	0	★	★	7
Kurahashi, 2007 [36]	★	★	0	★	★	★	★	★	7
Kurahashi, 2008 [35]	★	★	★	★	★	★	★	★	8
Lee, 2003 [37]	★	★	★	★	★	0	★	★	7
Lewis, 2009 [38]	★	★	0	★	★★	0	★	★	7
Li, 2008 [39]	★	★	★	★	★	0	★	★	7
Low, 2006 [40]	★	★	★	★	★★	★	★	★	9
Nagata, 2007 [41]	★	★	0	★	★	0	★	★	6
Nagata, 2016 [42]	★	★	0	★	★	★	★	★	7
Nomura, 2004 [43]	★	★	0	★	★	★	★	★	7
Ozasa, 2004 [44]	★	★	★	★	★	★	★	★	8
Park, 2008 [45]	★	★	0	★	★★	★	★	★	8
Severson, 1989 [46]	★	★	★	★	★	★	★	★	8
Sonoda, 2004 [47]	★	★	0	★	★	0	★	★	6
Strom, 1999 [48]	★	★	0	★	★★	0	★	★	7
Sung, 1999 [49]	★	★	0	★	★	0	★	★	6
Travis, 2009 [51]	★	★	★	★	★	★	★	★	8
Travis, 2012 [50]	★	★	★	★	★	★	★	★	8
Villenueve, 1999 [52]	★	★	★	★	★★	0	★	★	8
Wang, 2014 [53]	★	★	0	★	★★	★	★	★	8
Ward, 2008 [54]	★	★	★	★	★	★	★	★	8
Ward, 2010 [55]	★	★	0	★	★★	★	★	★	8
Wu, 2015 [56]	★	★	0	★	★★	★	★	★	8

^1^ Indicates that cases are independently validated for case-control studies (0, 1 star); ^2^ cases are from a representative population or drawn from the same community as the non-exposed cohort (0, 1); ^3^ community controls or structured interview for cohort studies (0, 1); ^4^ controls have no history of prostate cancer (endpoint) (0, 1); ^5^ study controls for most important factor (age) and family history of prostate cancer (0, 1, 2); ^6^ structured interview where blind to case/control status for case-control studies, record linkage or independent blind assessment for cohort studies (0, 1); ^7^ same method of ascertainment for cases and controls or follow-up was long enough for outcomes to occur (4 years) (0, 1); ^8^ same non-response rate or <20% lost to follow up (0, 1); ^9^ total: minimum equals 1; maximum equals 9 stars.
